# ASD and schizophrenia show distinct developmental profiles in common genetic overlap with population-based social communication difficulties

**DOI:** 10.1038/mp.2016.198

**Published:** 2017-01-03

**Authors:** B St Pourcain, E B Robinson, V Anttila, B B Sullivan, J Maller, J Golding, D Skuse, S Ring, D M Evans, S Zammit, S E Fisher, B M Neale, R J L Anney, S Ripke, M V Hollegaard, T Werge, iPSYCH-SSI-Broad Autism Group, A Ronald, J Grove, D M Hougaard, A D Børglum, P B Mortensen, M J Daly, G Davey Smith

**Affiliations:** 1 MRC Integrative Epidemiology Unit, University of Bristol, Bristol, UK; 2 School of Social and Community Medicine, University of Bristol, Bristol, UK; 3 Language and Genetics Department, Max Planck Institute for Psycholinguistics, Nijmegen, The Netherlands; 4 Analytic and Translational Genetics Unit, Massachusetts General Hospital and Harvard Medical School, Boston, MA, USA; 5 Stanley Center for Psychiatric Research and Medical and the Program in Medical and Population Genetics, Broad Institute of MIT and Harvard, Cambridge, MA, USA; 6 Centre for Child and Adolescent Health, University of Bristol, Bristol, UK; 7 Behavioural and Brain Sciences, Institute of Child Health, University College London, London, UK; 8 University of Queensland Diamantina Institute, Translational Research Institute, Brisbane, QLD, Australia; 9 MRC Centre for Neuropsychiatric Genetics and Genomics, Institute of Psychological Medicine and Clinical Neurosciences, Cardiff University, Cardiff, UK; 10 Donders Institute for Brain, Cognition and Behaviour, Radboud University, Nijmegen, The Netherlands; 11 Department of Psychiatry, Harvard Medical School, Boston, MA, USA; 12 Department of Psychiatry and Psychotherapy, Charité - Universitätsmedizin Berlin, Campus Mitte, Berlin, Germany; 13 Statens Serum Institut, Department of Congenital Disorders, Copenhagen, Denmark; 14 The Lundbeck Foundation Initiative for Integrative Psychiatric Research, iPSYCH, Aarhus, Denmark; 15 Institute of Biological Psychiatry, MHC Sct. Hans, Mental Health Services Copenhagen, Copenhagen, Denmark; 16 Institute of Clinical Sciences, Faculty of Medicine and Health Sciences, University of Copenhagen, Copenhagen, Denmark; 17 Department of Psychological Sciences, Birkbeck, University of London, London, UK; 18 Department of Biomedicine, Aarhus University, Aarhus, Denmark; 19 Centre for Integrative Sequencing, iSEQ, Aarhus University, Aarhus, Denmark; 20 Bioinformatics Research Centre, Aarhus University, Aarhus, Denmark; 21 National Centre for Register-based Research, Aarhus University, Aarhus, Denmark

## Abstract

Difficulties in social communication are part of the phenotypic overlap between autism spectrum disorders (ASD) and schizophrenia. Both conditions follow, however, distinct developmental patterns. Symptoms of ASD typically occur during early childhood, whereas most symptoms characteristic of schizophrenia do not appear before early adulthood. We investigated whether overlap in common genetic influences between these clinical conditions and impairments in social communication depends on the developmental stage of the assessed trait. Social communication difficulties were measured in typically-developing youth (Avon Longitudinal Study of Parents and Children, *N*⩽5553, longitudinal assessments at 8, 11, 14 and 17 years) using the Social Communication Disorder Checklist. Data on clinical ASD (PGC-ASD: 5305 cases, 5305 pseudo-controls; iPSYCH-ASD: 7783 cases, 11 359 controls) and schizophrenia (PGC-SCZ2: 34 241 cases, 45 604 controls, 1235 trios) were either obtained through the Psychiatric Genomics Consortium (PGC) or the Danish iPSYCH project. Overlap in genetic influences between ASD and social communication difficulties during development decreased with age, both in the PGC-ASD and the iPSYCH-ASD sample. Genetic overlap between schizophrenia and social communication difficulties, by contrast, persisted across age, as observed within two independent PGC-SCZ2 subsamples, and showed an increase in magnitude for traits assessed during later adolescence. ASD- and schizophrenia-related polygenic effects were unrelated to each other and changes in trait-disorder links reflect the heterogeneity of genetic factors influencing social communication difficulties during childhood versus later adolescence. Thus, both clinical ASD and schizophrenia share some genetic influences with impairments in social communication, but reveal distinct developmental profiles in their genetic links, consistent with the onset of clinical symptoms.

## Introduction

The phenotypic overlap between autism spectrum disorder (ASD) and schizophrenia is complex and dates back to Kanner in 1943.^1^ Individuals affected by either condition display deficits in the ability to initiate and maintain reciprocal interaction.^2^ This includes impairments in social cognition^3, 4^ but also poor social competence^5^ affecting verbal and nonverbal communication skills. Recent cross-disorder genetic analyses highlighted the continuity of psychiatric phenotypes beyond current diagnostic boundaries.^6^ The nature of shared genetic influences between childhood neurodevelopmental disorders, such as ASD, and adult-onset psychiatric illnesses, like schizophrenia, however, remains less well understood.

ASD represent a group of neurodevelopmental conditions with a typical age of onset before the age of 3 years affecting ~1 to 2% of children.^7, 8^ Core features include deficits in social interaction and communication, as well as highly restricted interests and/or stereotyped repetitive behaviours.^2^ By contrast, schizophrenia is an adult-onset psychiatric illness with a typical first-time diagnosis between 16 and 30 years. The disorder has a lifetime prevalence of ~1%^9^ and is characterised by hallucinations, delusions, disorganised speech or behaviour, apathy and lack of emotional reactivity.^2^ Both ASD and schizophrenia are highly heritable^10, 11^ and recent studies have linked different types of genetic variation including common variants,^10, 12, 13^ as well as rare inherited^14, 15^ and *de novo* variation^16, 17^ to risk of illness in both conditions. Contemporary research strongly supports a genetic overlap between ASD and schizophrenia for rare copy number variants^18^ and rare *de novo* mutation events^16^ with converging evidence for gene sets involved in synaptic function.^16^ The role of shared common genetic risk between ASD and schizophrenia, however, is less clear. Common genetic influences account for 25 to 33%^19^ of total liability to schizophrenia and up to 49% of total liability to ASD.^10, 12^ Despite this, the common genetic overlap between ASD and schizophrenia is small compared with the overlap between psychiatric adult-onset only disorders.^12, 20^

The framework of Research Domain Criteria (RDoC), including social communication difficulties, now actively facilitates the study of functional dimensions spanning the full range of human behaviour from normal to abnormal and across development.^21^ Common disorders, due to their polygenic architecture, can be understood as quantitative traits.^22^ For ASD, following the findings of earlier twin studies,^23, 24^ there is now molecular evidence for shared common genetic influences with social communication difficulties during childhood.^25^ The genetic continuity of social interaction and communication deficits in schizophrenia has not yet been observed though it can be hypothesised that such common genetic links exist given the impairments in social cognition within first-degree relatives of schizophrenia patients.^3^

Impaired abilities in social communication in affected children are heritable (twin*-h*^2^=0.74)^26^ and a large part of these genetic influences can be captured through common single-nucleotide polymorphisms (SNPs; SNP-*h*^2^⩽0.45).^27^ Beside some stable genetic influences,^28^ genetic factors underlying social interaction impairments and social communication difficulties vary during development,^27, 28^ especially for common variation.^27^ Thus, we hypothesise that also the genetic overlap between social communication difficulties and clinically recognised disorder may change during childhood and adolescence.

The primary aim of this study is to examine the nature of common polygenic influences in ASD and schizophrenia through their genetic overlap with phenotypic symptoms in the general population that are shared between both conditions, but differ according to developmental stage. We predict that if social communication difficulties are part of a common shared aetiology between ASD and schizophrenia, trait-disorder relationships for both conditions should follow similar patterns. Dissimilar patterns due to independent genetic influences would be expected for a non-shared genetic aetiology. Here, we report developmental profiles in common genetic overlap for both ASD and schizophrenia with respect to longitudinal measures of social communication difficulties within the general population. Analyses are based on the largest publicly available genome-wide data for ASD^29^ and schizophrenia,^13^ in addition to a large Danish ASD sample from the iPSYCH project and a deeply-phenotyped UK birth cohort, the Avon Longitudinal Study of Parents and Children (ALSPAC).^30, 31^

## Materials and methods

### Genome-wide summary statistics

#### Population-based social communication difficulties

Genome-wide association studies (GWASs) were carried out in ALSPAC participants, a UK population-based longitudinal pregnancy-ascertained birth cohort (estimated birth date: 1991–1992).^30, 31^ Ethical approval was obtained from the ALSPAC Law-and-Ethics Committee (IRB00003312) and the Local Research-Ethics Committees, written informed consent was obtained from a parent or individual with parental responsibility and assent was obtained from child participants.

ALSPAC children were genotyped using the Illumina HumanHap550 quad-chip and imputation was performed on 8237 children and 477,482 SNP genotypes using a 1000 Genomes reference (PhaseI_v3, http://www.1000genomes.org/)^32^ (Supplementary Methods).

Quantitative social communication problems in ALSPAC children were assessed with the 12-item Social Communication Disorder Checklist (SCDC; score-range: 0 to 24).^26^ The SCDC is a brief screening instrument of social reciprocity and verbal/nonverbal communication (for example, ‘Not aware of other people’s feelings’), with high reliability and good validity,^26^ which has been extensively investigated.^26, 27, 33^ Higher SCDC scores reflect more social communication deficits and are positively skewed (Supplementary Figure 1). Mother-reported scores for children and adolescents were repeatedly measured at 8, 11, 14 and 17 years (Supplementary Table 1) and are inter-correlated (Spearman's *ρ*: 0.39 to 0.57, Supplementary Table 2). Information on phenotypic and genotypic data was available for 4175 to 5553 children (Table 1).

SCDC scores were residualised for sex, age and the two most significant ancestry-informative principal components^34^ and then rank-transformed (Supplementary Figure 2). Transformed scores showed similar correlation patterns as untransformed scores (Pearson' *r*: 0.38 to 0.61, Supplementary Table 2).

Genome-wide single marker summary statistics were generated by regressing rank-transformed residuals on allele dosages using SNPTEST^35^ (without genomic control-based correction^36^).

#### Clinical ASD

The Psychiatric Genomics Consortium (PGC) has completed a genome-wide scan of 5305 ASD cases and their parents (PGC-ASD), all of European ancestry (2015 freeze; summary results at http://www.med.unc.edu/pgc/). An ASD diagnosis was confirmed using research standard diagnoses and expert clinical consensus diagnoses. 94.1% of all ASD cases had also a diagnosis of autism from the Autism Diagnostic Interview-Revised^37^ and/or the Autism Diagnostic Observation Schedule.^38^ Genome-wide data were imputed to a 1000 Genomes reference (PhaseI_v3) and genetic association studied using a case and pseudo-control design.^29^ This design is robust to population stratification as pseudo-controls are based on un-transmitted parental alleles, and thus cases and pseudo-controls are ancestrally matched. To replicate findings, we analysed ASD GWAS summary results in the Danish iPSYCH project (iPSYCH-ASD: 7783 ASD cases, 11 359 controls) using samples from the Danish Neonatal Screening Biobank hosted by Statens Serum Institute (Supplementary Methods). The iPSYCH-ASD project aims to genotype all Danish individuals with available DNA from bloodspots and an ASD diagnosis (International Classification of Diseases^39^) in their medical record. iPSYCH-ASD has been genotyped using the Illumina Infinium PsychArray BeadChip and genotypes were imputed to a 1000 Genomes template (PhaseI_v3). This study has been approved by the Danish research ethical committee system.

Note that also a small number of ALSPAC children with clinical ASD (N⩽ 36) has been included in this study (Supplementary Methods).

#### Clinical schizophrenia

A large PGC mega-analysis on schizophrenia has been carried out studying individuals of predominantly European descent^13^ (Summary results at http://www.med.unc.edu/pgc/). Cases met diagnostic criteria for either schizophrenia or schizoaffective disorder.^13^ Here, we investigated two non-overlapping schizophrenia subsets: (1) PGC-SCZ1 (11 958 cases, 12 710 controls), constructed as part of the first PGC mega-analysis of schizophrenia,^13, 40^ and (2) PGC-SCZ2i, containing novel PGC-SCZ2 cases and controls not included in PGC-SCZ1 (22 283 cases, 32 894 controls, 1235 trios).^13^ In addition, we studied the combined PGC-SCZ2 sample (PGC-SCZ1+PGC-SCZ2i: 34 241 cases, 45 604 controls, 1235 trios) of the second PGC mega-analysis of schizophrenia.^13^ As PGC-SCZ2 contains 1836 cases and 3383 controls from East Asia, we also studied a PGC-SCZ2 sample of European ancestry only (PGC-SCZ2-Eur: 32 405 cases, 42 221 controls, 1235 trios). Genome-wide data were imputed to a 1000 Genomes template (PhaseI_v3).

The studied population-based and clinical samples (Table 1) contain no sample overlap.

#### Other adult-onset disorders

To analyse the specificity of genetic overlap between SCDC scores and schizophrenia, we studied further adult-onset psychiatric disorders, such as major depressive disorder (MDD) and bipolar disorder (BIP; Supplementary Methods).

### Statistical methods

Linkage disequilibrium (LD) score regression^41^ was applied to estimate the cumulative effect of common SNPs on either variation in developmental SCDC scores or risk to disorder (SNP-*h*^2^), using GWAS statistics and exploiting LD patterns in the genome. LD score correlation^20^ analysis was carried out to estimate genetic correlations (r_g_) between SCDC scores and clinical conditions, or among clinical conditions, that is, the extent to which two phenotypes share common genetic factors, based on GWAS statistics. All analyses were performed with LDSC software^20, 41^ using HapMap3 markers^42^ (Supplementary Methods).

Polygenic risk scores (PGS)^43, 44^ were analysed to estimate the explained phenotypic variance in social communication difficulties due to risk-increasing alleles for clinical disorder. Using a range of *P*-value thresholds (0.001<*P*_T_⩽ 1), PGS for ASD (based on PGC-ASD), schizophrenia (based on PGC-SCZ2) and schizophrenia subsamples (based on PGC-SCZ1 and PGC-SCZ2i) were generated in ALSPAC (Table 1) using imputed genotypes (1000 Genomes, PhaseI_v3, INFO>0.8). For this, common autosomal signals observed in clinical samples (with MAF>0.01 in ALSPAC) were clumped (LD-r^2^>0.25, ±500 kb) consistent with current guidelines^45^ using PLINK,^46^ excluding duplicate SNPs (Supplementary Methods). Rank-transformed SCDC scores were regressed on Z-standardised PGS (Ordinary least square regression, R software Rv3.2.2, https://cran.r-project.org/), and the proportion of phenotypic variance explained by each PGS predictor reported as adjusted regression *R*^2^. Note that assuming an infinitely large clinical 'discovery' sample, the regression *R*^2^ is equivalent to the product of r_g_ squared and the heritability of the explained Z-standardised trait.^44^

Mixed Poisson regression (R:lme4 library) was utilised to test the trend in common genetic overlap longitudinally using untransformed SCDC scores. Repeatedly assessed SCDC score counts were regressed on ASD- and schizophrenia-PGS with overdispersion being accounted for through the random error part.^47^ Models included fixed effects for ASD-PGS and schizophrenia-PGS, sex, age at assessment, as well as random intercepts. Beta-coefficients for PGS quantify here the increase in natural-log SCDC scores for each increase in one standard deviation of PGS. Differences in sample-dropout across time were accounted for through bootstrapping, generating parametric 95%-bootstrap confidence intervals (*N*_Bootstrap_=500). We have not estimated adjusted *R*^2^-related measures due to the difficulty of defining the residual variance for non-Gaussian responses, especially within a mixed model context.^48^

Genome-wide Complex Trait Analysis (GCTA)^49, 50^ was utilised to estimate SNP-*h*^2^ and genetic correlations among SCDC scores, as published previously,^27^ for comparison only (Supplementary Methods).

Attrition analysis in ALSPAC studied the relationship between SCDC-missingness at each assessed age and PGS for clinical ASD and schizophrenia (Supplementary Methods).

## Results

### SNP-heritabilities for social communication difficulties and psychiatric disorder

Genome-wide analyses of population-based SCDC scores at 8, 11, 14 and 17 years provided little evidence for bias in GWAS statistics due to population stratification. The estimated LDSC-*h*^2^ intercepts were consistent with one, ranging from 0.988 (s.e.=0.0067) to 1.009 (s.e.=0.0070; Table 2). In subsequent analyses LDSC-*h*^2^ intercepts were thus constrained to one, including LDSC correlation analyses.

Cumulative influences of SNPs on variation in SCDC scores were strongest at the age of 8, 11 and 17 years with LDSC-*h*^2^ estimates of 0.19 (s.e.=0.06), 0.17 (s.e.=0.07) and 0.30 (s.e.=0.11), respectively (Table 2). The estimates were lower, however, at 14 years (LDSC-*h*^2^=0.08 (s.e.=0.06)). These LDSC-based findings mirrored closely GCTA-*h*^2^ estimates using GREML (Table 2), although latter might potentially be biased.^51^ SCDC scores shared furthermore genetic factors across development (GREML r_g_=0.38 (s.e.=0.16) to 0.95 (s.e.=0.34), *P*_min_=2 × 10^−7^), as previously reported,^27^ with lower correlations across wider age gaps (Supplementary Table 3).

A common genetic basis for ASD has been described earlier^12, 25^ including PGC-ASD (liability-scale LDSC-*h*^2^=0.23 (s.e.=0.03))^25^ and iPSYCH-ASD (liability-scale LDSC-*h*^2^=0.14 (s.e.=0.03)),^25^ with strong evidence for similar polygenic architectures among samples (r_g_=0.74 (s.e.=0.07), *P*<10^−20^).^25^ Also, it is known^12, 13, 40^ that common genetic factors influence schizophrenia liability. Liability-scale LDSC-SNP-*h*^2^ estimates for PGC-SCZ1, PGC-SCZ2i, PGC-SCZ2Eur and PGC-SCZ2 were 0.31 (s.e.=0.02), 0.24 (s.e.=0.01), 0.25 (s.e.=0.01) and 0.25 (s.e.=0.01), respectively (assumed population-prevalence of 0.01), with strong evidence for shared genetic factors among independent samples (PGC-SCZ1 and PGC-SCZ2i: r_g_=0.96 (s.e.=0.024), *P*<10^−20^).

### Genetic correlations between social communication difficulties and psychiatric disorder

**Figure 1:**
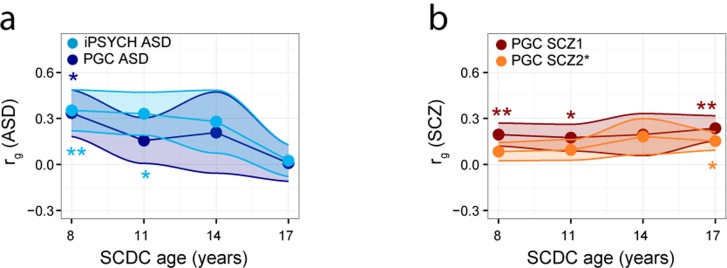
Genetic correlations between (**a**) clinical ASD and (**b**) clinical schizophrenia and SCDC scores during development. Genetic correlations between clinical disorder and rank-transformed SCDC scores in ALSPAC (at 8, 11, 14 and 17 years) were estimated cross-sectionally using LD-score correlation analysis^20^ and are shown with their standard errors (shaded). Standard error distributions for SCDC scores across age were approximated using loess. *P*-values indicate the probability that the true genetic correlation is different from zero (*P⩽0.05, **P⩽0.01). ALSPAC, Avon Longitudinal study of Parents and Children; ASD, autism spectrum disorder; iPSYCH-ASD, iPSYCH-SSI-BROAD Autism project; LD, linkage disequilibrium; PGC, Psychiatric Genomics Consortium; PGC-ASD, ASD collection of the PGC; PGC-SCZ1, Samples of the first PGC mega-analysis of SCZ; PGC-SCZ2i, PGC-SCZ2 samples not analysed within PGC-SCZ1; SCDC, Social Communication Disorder Checklist; SCZ, schizophrenia.

As part of a two-stage analysis design (Table 1), we used constrained LD score correlation to study the genetic overlap between psychiatric disorder and social communication problems during development. Genetic correlations between rank-transformed social communication difficulties and clinical ASD decreased in point estimates with progressing age of the trait (Figure 1a, Supplementary Table 4). For PGC-ASD, the genetic link with SCDC scores was strongest at 8 years (r_g_=0.34 (s.e.=0.15), *P*=0.027)^25^ and attenuated by 17 years (r_g_=0.01 (s.e.=0.12), *P*=0.94). This pattern was replicated in iPSYCH-ASD (r_g_=0.35 (s.e.=0.13), *P*=0.008 and r_g_=0.02 (s.e.=0.10), *P*=0.81, respectively, Supplementary Table 4). In contrast, common genetic links between schizophrenia and social communication difficulties during childhood and adolescence persisted and increased in point estimates (Figure 1b). Within PGC-SCZ1, genetic overlap with SCDC scores started to emerge at 8 years (r_g_=0.20 (s.e.=0.08), *P*=0.01) and was strongest at 17 years (r_g_=0.24 (s.e.=0.08), *P*=0.004; Figure 1b, Supplementary Table 4). The genetic link during later adolescence was replicated in PGC-SCZ2i (age 17: r_g_=0.15 (s.e.=0.06), *P*=0.011, Figure 1b) and also observed in the combined PGC-SCZ2 sample (PGC-SCZ1+PGC-SCZ2i: r_g_=0.18 (s.e.=0.06), *P*=0.003, Supplementary Table 4). These findings were not affected by the presence of a small proportion of individuals of Asian origin (PGC-SCZ2-Eur: r_g_=0.18 (s.e.=0.06), *P*=0.004, Supplementary Table 4). Importantly, other PGC adult-onset disorders, such as MDD and BIP, showed no correlations with SCDC scores (Age 17: MDD-r_g_=−0.05 (s.e.=0.11), *P*=0.65 and BIP-r_g_=0.04 (s.e.=0.08), *P*=0.62, Supplementary Table 4) suggesting that findings are specific to schizophrenia. Note that LD-score correlations between schizophrenia and ASD (r_g_=0.20 (s.e.=0.05), *P*=0.00011) were modest, compared with considerably stronger links between schizophrenia and other adult-onset disorders (for example, BIP-r_g_=0.76 (s.e.=0.04), *P*=6.5 × 10^−70^, Supplementary Table 5), as previously reported.^20^

For comparison, we also analysed trait-disorder overlap using LD score correlation without constraining intercepts (Supplementary Table 4). In the presence of genetic links, unconstrained r_g_-point estimates were, overall, in close correspondence with constrained estimates, but had wider standard errors.

### Polygenic scores for risk-increasing alleles predicting social communication difficulties

**Figure 2:**
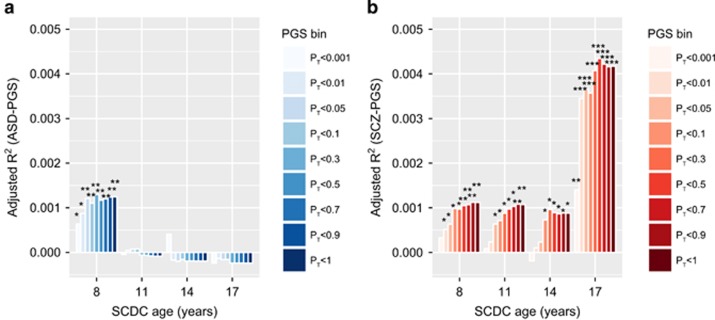
Proportion of variance in SCDC scores explained by polygenic scores for (**a**) clinical ASD and (**b**) clinical schizophrenia. Polygenic scores were constructed in ALSPAC based on the largest publicly available samples for ASD (PGC-ASD) and schizophrenia (PGC-SCZ2) as a training set, and then Z-standardised. The proportion of explained phenotypic variance in rank-transformed SCDC scores (adjusted regression *R*^2^) is displayed cross-sectionally at 8, 11, 14 and 17 years and given with respect to ASD-PGS (**a**) and schizophrenia-PGS (**b**). Nine different *P*-value thresholds *P*_T_ for selecting risk alleles (PGS bins) in clinical samples are displayed. Starred *P*-values indicate the strength of the association (*P⩽0.05, **P⩽0.01). ALSPAC, Avon Longitudinal study of Parents and Children; ASD, autism spectrum disorder; PGC-ASD, ASD collection of the PGC; PGC, Psychiatric Genomics Consortium; PGC-SCZ2, Samples of the second PGC mega-analysis of SCZ; PGS, polygenic scores; PGS bin, Z-standardised polygenic scores according to threshold *P*_*T*_; SCDC, Social Communication Disorder Checklist; SCZ, schizophrenia.

To provide an absolute measure of shared genetic influences between traits and clinically recognised conditions, we assessed the phenotypic variance in rank-transformed social communication difficulties due to risk-increasing alleles using polygenic scoring^43, 44^ (Table 1). Alleles more common in ASD cases than in pseudo-controls were only associated with variation in SCDC scores at 8 years (PGC-ASD: adjusted *R*^2^_max_=0.13%, *P*_min_=0.0042, Figure 2a, Supplementary Table 6). In contrast, alleles more often present in schizophrenia cases than controls explained predominantly variation in social communication difficulties at 17 years, based on risk alleles in both PGC-SCZ subsamples (PGC-SCZ1: adjusted *R*^2^_max_=0.26%, *P*_min_=0.00058; PGC-SCZ2i: adjusted *R*^2^_max_=0.19%, *P*_min_=0.0028; Supplementary Table 7) and the combined PGC-SCZ2 sample (adjusted *R*^2^_max_=0.43%, *P*_min_=0.000012, Figure 2b, Supplementary Table 6). Excluding ALSPAC children with a clinical ASD diagnosis had little influence on the reported changes in genetic effect (Supplementary Table 8). Importantly, adjustment of ASD-PGS and schizophrenia-PGS for each other did not affect the nature of these findings, suggesting the independence of ASD- and schizophrenia-related polygenic influences (Supplementary Table 6).

**Figure 3:**
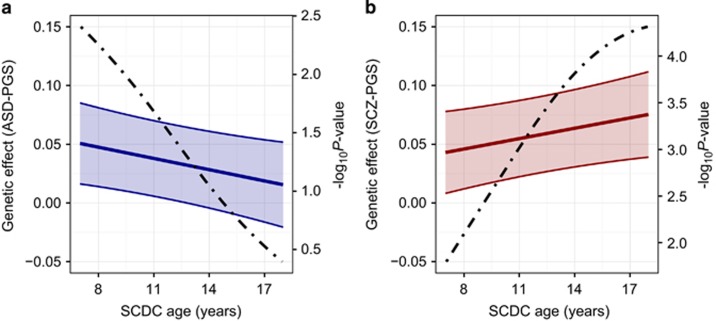
Developmental changes in genetic effects of polygenic scores for (**a**) clinical ASD and (**b**) clinical schizophrenia on SCDC scores. Polygenic scores (PGS) were constructed in ALSPAC based on the largest publicly available samples for ASD (PGC-ASD) and schizophrenia (PGC-SCZ2) as a training set, and then Z-standardised. A *P*-value threshold of *P*_*T*_ <0.05 for selecting risk alleles in clinical samples is displayed. Using a mixed Poisson regression framework, longitudinal measures of untransformed SCDC score counts were regressed on ASD-PGS and schizophrenia-PGS simultaneously allowing for changes in genetic effects over time. Repeatedly assessed SCDC score counts in ALSPAC were available at 8, 11, 14 and 17 years of age with individual ages ranging between 7 to 18 years. Genetic effects for ASD-PGS (**a**) and their 95% confidence intervals (shaded) as well as schizophrenia-PGS (**b**) and their 95% confidence intervals (shaded) were estimated across development, and show the increase in SCDC log counts per standard deviation in PGS score. A dotted line indicates the *P*-value of the genetic effect. ALSPAC, Avon Longitudinal study of Parents and Children; ASD, autism spectrum disorder; PGC-ASD, ASD collection of the PGC; PGC, Psychiatric Genomics Consortium; PGC-SCZ2, Samples of the second PGC mega-analysis of SCZ; SCDC, Social Communication Disorder Checklist; SCZ, schizophrenia.

To assess developmental trends in common genetic trait-disorder overlap, we modelled the effect of ASD-PGS and schizophrenia-PGS on untransformed SCDC scores longitudinally. Applying a mixed Poisson model, we found evidence for age-specific changes in genetic effects for both ASD-PGS and schizophrenia-PGS (Supplementary Table 9). For example, at *P*_T_<0.05 (Figure 3), a threshold shown to predict schizophrenia case-ness in independent samples,^13^ the effect of ASD-PGS decreased with progressing age of the trait (ASD-PGS × SCDC-age: Beta=−0.0031 (s.e.=0.0014), *P*=0.019, 95%-bootstrapped confidence interval: −0.0057 to −0.00035), while the effect of schizophrenia-PGS increased (schizophrenia-PGS x SCDC-age: Beta=0.0029 (s.e.=0.0014), *P*=0.030, 95%-bootstrapped confidence-interval: 0.00047 to 0.0054). Consistent with the findings for rank-transformed scores, ASD-related polygenic influences on SCDC score counts were strongest during childhood (age 8: Beta=0.047 (s.e.=0.017), *P*=0.0056; age 17: Beta=0.019 (s.e.=0.018), *P*=0.29), while schizophrenia-related polygenic effects were more pronounced during later adolescence (age 8: Beta=0.046 (s.e.=0.017), *P*=0.0080; age 17: Beta=0.072 (s.e.=0.018), *P*=0.000056). Similar developmental changes in genetic overlap were also found for other PGS thresholds (Supplementary Table 9).

### Attrition in ALSPAC

Analyses of SCDC-missingness in ALSPAC were carried out to investigate potential sources of bias (Supplementary Table 10). Using for simplicity a PGS threshold of *P*_T_<0.05, there was little evidence for a relationship between sample-dropout and ASD-PGS, especially after adjustment for maternal educational level (age 8: odds ratio=0.99 (s.e.=0.03), *P*=0.82), although there was support for an association with schizophrenia-PGS (age 17: odds ratio=1.10 (s.e.=0.03), *P*=0.000050), consistent with previous studies.^52^

## Discussion

This study provided evidence for shared common genetic overlap between social communication difficulties and both ASD and schizophrenia, but does not imply a shared genetic susceptibility between these clinical conditions. Instead, we identified distinct patterns in genetic trait-disorder relationships, largely consistent with the onset of clinical symptoms. Genetic links were driven by independent polygenetic influences and showed opposite trends in magnitude with progressing age of the population-based trait, as supported by longitudinal analyses.

Genetic overlap with ASD was strongest for social communication difficulties during middle childhood (r_g_~33%), in line with recent cross-sectional studies,^25^ while those with schizophrenia was strongest for social communication difficulties during later adolescence (r_g_~18%). Complementary estimates were provided by polygenic scoring analyses. Up to 0.13% phenotypic variation in social communication difficulties could be explained by ASD risk-increasing alleles during childhood and up to 0.43% phenotypic variation by schizophrenia risk-increasing alleles during later adolescence, independently of each other. The genetic overlap with social communication difficulties during later adolescence was not observed for other adult-onset disorders, such as BIP, despite their strong genetic links with psychosis,^12^ making unspecific age-related influences unlikely. Thus, our findings suggest that social communication impairments are part of the genetically influenced phenotypic spectrum of schizophrenia.

Changes in genetic overlap over time need to be viewed within the context of cohort-specific sampling properties and clinical sample power. For instance, it is possible that the genetic overlap between schizophrenia and social communication difficulties has been underestimated, as SCDC-missingness, and more generally study non-participation,^52^ has been related to common genetic risk for schizophrenia. In contrast, there was little evidence for a link between SCDC missingness and common ASD risk. In addition, mother-report of social communication difficulties may have contributed to enhanced variance sharing among population-based traits, and thus underestimated the true variation in child genetic effects. Finally, the studied clinical discovery sets differed in their inherent power. For example, the power^44^ of ASD-PGS (PGC-ASD ~5000 trios) was only 0.58 compared with 0.99 for schizophrenia-PGS (PGC-SCZ2, *N*~80 000), assuming a liability-scale SNP-*h*^2^ of 0.23 for ASD and 0.25 for schizophrenia, a disease prevalence of 0.01, a type-I-error rate of 0.05 and a population-based target sample of 5000 individuals. Under attrition, such as a decrease of ~1000 ALSPAC participants during later adolescence, the power of ASD-PGS would further drop (to 0.49), while the power of schizophrenia-PGS remains largely unaffected (0.99). Longitudinal analyses, adjusting for differences in population-based sample numbers across time through bootstrapping, suggested, however, that the observed developmental changes in polygenic risk effects are robust, even in the presence of sample dropout.

Our results have direct relevance for the definition of RDoC^21^ within a developmental context. The lack of support for shared polygenic effects between ASD and schizophrenia, with respect to social communication impairments, is in agreement with recent studies. Molecular analyses of PGC samples reported modest correlations between ASD and schizophrenia^20, 29^ (r_g_~0.20), confirmed within this study, and twin research suggested little genetic overlap between autistic traits and psychotic experiences.^53^ The absence of shared aetiological factors strengthens furthermore positions suggesting that the exact nature of social deficits implicated within ASD and schizophrenia differs from each other.^54^ Here we show that common genetic variation underlying complex disorders can be dissected through temporal changes in the genetic architecture of behavioural symptoms that are shared between disorders. Thus, a developmental analysis of genetic relationships between population-based and clinical samples can be informative with regard to the dimensional nature of psychiatric illness without discarding the aetiology of different disorders, a concern often raised with respect to RDoC.^21^

The identification of distinct patterns in common genetic overlap between social communication difficulties and psychiatric illness is consistent with the presence of multiple distinct genetic influences contributing to variation in social communication behaviour during development. While genetic factors underlying SCDC scores across ~3-year intervals are stable and shared by at least 80%, only ~50% of common genetic influences are shared across 10 years intervals.^27^ This may suggest developmental (but not rapid) changes in the phenotype capture by the SCDC with progressing age. For example, it is possible that the behavioural phenotypes influencing SCDC scores at age 8 or 11 years are, in terms of average composition, different from those influencing the scale at age 17. Social communication abilities comprise many components, such as social interaction, social cognition, pragmatic and language processing skills (http://www.asha.org/), some of which will vary during child and adolescent development, including changes in the social-cognitive understanding of friendship and peer interaction.^55^ One might envisage that these phenotypic changes reflect distinct genetic factors driving different stages of postnatal brain development.^56^ In addition, social communication difficulties have been linked to behavioural problems.^57^ Note that the SCDC has a high sensitivity but a lower specificity in discriminating ASD from the non-ASD patients in the presence of other clinical disorders.^26^ Thus, the SCDC is likely to capture multiple behavioural and cognitive dimensions related to social communication problems during the course of child and adolescent development, spanning around 10 years, which give rise to distinct patterns in trait-disorder overlap. This poses questions on the nature of genetic influences affecting variation in social communication impairments across development that will require exploration with longitudinal genome-wide approaches and biological network analyses.

## Conclusions

Social communication difficulties are phenotypically shared with both ASD and schizophrenia and show common genetic overlap with both disorders. These polygenic links manifest, however, as distinct developmental profiles and do not imply a shared genetic susceptibility between these clinical conditions.

## Figures and Tables

**Table 1:** Genome-wide summary statistics

*Sample*	*Source*	*Phenotype/diagnosis*	*Ethnicity*	N	*Study design*	
					*LD score correlation*	*PGS*
ALSPAC	General population	Mother-reported SCDC scores	White European	5553 (8 years) 5462 (11 years) 5060 (14 years) 4175 (17 years)	r_g_ with respect to SCDC scores	Polygenic effect of risk-increasing alleles on SCDC scores^a^
PGC-ASD	Clinical sample	ASD	White European	5305 cases; 5305 pseudo-controls	Discovery sample	Discovery sample^b,c^
iPSYCH-ASD	Clinical sample	ASD	White European	7783 cases; 11 359 matched controls	Replication sample	—
PGC-SCZ1	Clinical sample, subset of PGC-SCZ2	Schizophrenia or schizoaffective disorder	White European	11 958 cases; 12 710 controls	Independent sample	Independent sample^c^
PGC-SCZ2i	Clinical sample, subset of PGC-SCZ2	Schizophrenia or schizoaffective disorder	Predominantly white European	22 283 cases; 32 894 controls, 1235 trios (including 1836 cases and 3383 controls of East Asian ancestry)	Independent sample	Independent sample^c^
PGC-SCZ2	Clinical sample	Schizophrenia or schizoaffective disorder	Predominantly white European	34 241 cases; 45 604 controls, 1235 trios (including 1836 cases and 3383 controls of East Asian ancestry)	Combined sample	Combined sample^b,c^
PGC-SCZ2-Eur	Clinical sample	Schizophrenia or schizoaffective disorder	White European	32 405 cases; 42 221 controls, 1235 trios	Combined sample^d^	—

Abbreviations: ALSPAC, Avon Longitudinal study of Parents and Children; ASD, autism spectrum disorder; iPSYCH-iPASD, iPSYCH-SSI-BROAD Autism project; LD, linkage disequilibrium; PGC, Psychiatric Genomics Consortium; PGC-SCZ1, Samples of the first PGC mega-analysis of SCZ; PGC-SCZ2i, PGC-SCZ2 samples not analysed within PGC-SCZ1; PGC-SCZ2, Samples of the second PGC mega-analysis of SCZ (PGC-SCZ1+PGC-SCZ2i); PGC-SCZ2-Eur, PGC-SCZ2 participants of European ancestry only (exclusion of 1836 cases and 3383 controls from East Asia);^13^ PGS, polygenic scores; r_g_, genetic correlation; SCDC, Social Communication Disorder Checklist; SCZ, schizophrenia.

All samples were imputed to a 1000 genomes reference (Phase1_v3); Note that there is no overlap between population-based and clinical samples.

a ALSPAC is target sample.

b Largest publicly available set of genome-wide summary statistics.

c Sensitivity analysis.

d Clinical samples are training sets.

**Table 2:** LD-score regression and GCTA results for SCDC scores in ALSPAC

*SCDC score*	*LD score regression*					*GCTA*^a^	
	*Unconstrained*^b^	*Constrained*^c^	*λ*_*GC*_	*Mean* χ^*2*^	N	h^*2*^*(SE)*^a^	N^d^
	*Intercept(SE)*	h^*2*^*(SE)*					
8 y	0.992 (0.0067)	0.19 (0.06)	1.023	1.022	5553	0.24 (0.07)	5137
11 y	1.000 (0.0065)	0.17 (0.07)	1.014	1.019	5462	0.17 (0.07)	5058
14 y	0.988 (0.0067)	0.08 (0.06)	1.005	1.009	5060	0.08 (0.07)	4735
17 y	1.009 (0.0070)	0.30 (0.11)	1.029	1.025	4175	0.45 (0.08)	3978

Abbreviations: ALSPAC, Avon Longitudinal study of Parents and Children; GCTA, genome-wide complex trait analysis; *h*^2^, SNP heritability; LD, linkage disequilibrium; SCDC, Social Communication Disorder Checklist; y, age at assessment in years; λ_GC_, Genomic inflation factor.

a Findings correspond closely to previously published estimates.^27^

b LD score regression using an unconstrained intercept.

c LD score regression constraining the intercept for the SNP-*h*^2^ estimation to one.

d Differences compared with the total sample *N* are due to the exclusion of individuals with a relatedness of ≥2.5%.

## Supplementary Material

Supplemenetary Material
