# Supplementary material for: ASD and schizophrenia show distinct developmental profiles in common genetic overlap with population-based social communication difficulties
Source: Mol Psychiatry. 2017 Jan 3;23(2):263–70. doi: 10.1038/mp.2016.198 (PMC5382976; doi:10.1038/mp.2016.198)
Supplement: Supplemenetary Material [file mp2016198x1.docx]

Supplementary information

**ASD and schizophrenia show distinct developmental profiles in common genetic overlap with population-based social-communication difficulties**

Contents

**Supplementary methods**

- ALSPAC genotyping quality control
- iPSYCH ASD sample
- ASD cases within ALSPAC
- Other adult-onset disorders
- Linkage-disequilibrium(LD)-score regression and correlation
- Polygenic scoring analysis
- Genome-wide Complex Trait Analysis
- Attrition within ALSPAC

**Supplementary references**

**iPSYCH‐SSI‐Broad Autism Group**

**Web resources**

**Supplementary tables**

- Supplementary Table 1: Descriptives of SCDC scores
- Supplementary Table 2: Phenotypic correlations of SCDC scores
- Supplementary Table 3: Genetic correlations of SCDC scores
- Supplementary Table 4: Genetic correlations between psychiatric disorders and SCDC scores
- Supplementary Table 5: Genetic correlations between schizophrenia and other psychiatric disorders
- Supplementary Table 6: Association of ASD and schizophrenia risk scores with SCDC scores
- Supplementary Table 7: Association of schizophrenia risk scores with SCDC scores (PGC-SCZ2 subsamples)
- Supplementary Table 8: Association of ASD and schizophrenia risk scores with SCDC scores (exclusion of ALSPAC ASD cases)
- Supplementary Table 9: Association of ASD and schizophrenia risk scores with SCDC scores (longitudinal analysis of effect changes)
- Supplementary Table 10: Association of ASD and schizophrenia risk scores with SCDC-missingness

**Supplementary figures**

- Supplementary Figure 1: Distribution of raw SCDC scores in ALSPAC
- Supplementary Figure 2: Distribution of rank-transformed SCDC scores in ALSPAC

**Supplementary methods**

ALSPAC genotyping quality control

Children from the Avon Longitudinal study of Parents and Children (ALSPAC) were genotyped using the Illumina HumanHap550 quad chip genotyping platforms by 23andme subcontracting the Wellcome Trust Sanger Institute, Cambridge, UK and the Laboratory Corporation of America, Burlington, NC, US. After quality control (individual call rate>0.97, SNP call rate>0.95, Minor allele frequency (MAF)>0.01, Hardy-Weinberg equilibrium (HWE) P>10^-7^, and removal of individuals with cryptic relatedness and non-European ancestry, 8,237 children and 477,482 SNP genotypes were retained. SNPs were flipped to the forward strand and haplotypes were estimated using ShapeIT (v2.r644)^1^. Imputation was performed using Impute V2.2.2^2^ in 1000 genomes reference haplotypes (Version 1 Phase 3). Minor changes with respect to previously reported sample numbers are due to withdrawn consents and alterations in GWAS quality control^3^.

iPSYCH ASD sample

The iPSYCH-ASD (7,783 ASD cases, 11,359 controls, waves 1-10) is a new case-control ASD sample derived from the Danish Bloodspot resource. In 1981, Denmark began storing neonatal bloodspots and collected samples have been subsequently linked to the Danish Psychiatric Central Register. DNA extracted from the bloodspots can be successfully amplified and employed in GWAS^4,5^. The iPSYCH-ASD project aims to genotype all Danish individuals with available DNA from bloodspots and an ASD diagnosis in their medical record (International Classification of Diseases codes: F84.0, F84.1, F84.5, F84.8, and F84.9)^6^. iPSYCH-ASD has been genotyped using the Illumina Infinium PsychArray BeadChip and genotypes were imputed to a 1000 Genomes template (PhaseI_v3). Genotyping, quality control, imputation and genetic association analysis were carried out by the PGC Statistical Analysis Group and the iPSYCH-ASD Analysis Group using the Ricopili pipeline with standard PGC settings^7^.

ASD cases within ALSPAC

The ALSPAC cohort includes a small proportion of children with clinical ASD, who were identified from either National Health Service community paediatric records or Education Service databases for the region^8^. Eighty-six children with autism spectrum disorder were identified by age 11, giving a prevalence of 62 per 10,000 children. Twenty to 36 children had genotypic and phenotypic information available at 8, 11, 14 and 17 years and were unrelated and of white European origin.

Other adult-onset disorders

To exclude the possibility that identified genetic overlaps between Social-Communication Disorder Checklist (SCDC) scores and schizophrenia are due to unspecific age-related effects rather than disorder-specific effects (given the later age of onset of symptoms in schizophrenia), we also investigated other adult-onset psychiatric disorders studied by the Psychiatric Genomic Consortium (PGC), such as major depressive disorder (MDD)^9^ and bipolar disorder (BIP)^10^.

The PGC conducted a mega-analysis for MDD in a large sample of patients and controls (PGC-MDD: 9,240 MDD cases and 9,519 controls; summary results at <http://www.med.unc.edu/pgc/>), all of European descent^9^. Cases were required to have a diagnosis of DSM-IV lifetime MDD^9^. Genotypes were imputed to combined HapMap phase 3 CEU and TSI samples^9^.

In addition, a PGC mega-analysis on BIP (PGC-BIP: 7,481 cases, 9,250 controls; summary results at <http://www.med.unc.edu/pgc/>) has been carried out studying also individuals of European ancestry ^10^. Cases had either a clinical diagnosis of bipolar disorder type 1, bipolar disorder type 2, schizoaffective disorder bipolar or another bipolar diagnosis. Genotypes were imputed to a HapMap phase 2 CEU reference sample^10^.

Linkage-disequilibrium(LD)-score regression and correlation

*Linkage-disequilibrium(LD)-score regression*^11^ was applied to estimate the cumulative effect of common SNPs (SNP-h^2^) to variation in developmental SCDC scores, using GWAS statistics and exploiting LD patterns in the genome (LDSC-h^2^). LD-score regression distinguishes confounding biases from polygenic influences in genome-wide analyses^11^. It exploits the fact that for a polygenic trait variants in LD with many other markers have, on average, higher test-statistics (e.g. χ^2^) than variants in LD with only a few variants^11^. To estimate LDSC-h^2^, genome-wide χ^2^-statistics are regressed on the amount of genetic variation captured by each SNP (LD-score). The intercept of this regression minus one is an estimator of the mean contribution of confounding bias to the inflation in the mean χ^2^-statistic^11^. In extension, *LD-score correlation*^12^ analysis can be carried out to estimate the genetic correlation (r_g_ ;ranging from -1 to 1) between common variants in distinct samples by regressing the product of test statistics against the same LD-score. All analyses were performed with LDSC software^11,12^ and based on the set of well-imputed HapMap3 SNPs^13^ and a European reference panel of LD-scores^12^. Note that LDSC correlation analysis does not yet allow estimating genetic correlations longitudinally and independently of each other. For constrained LDSC regression analysis, we constrained the intercept for SNP-h^2^ estimations to one, given there is little evidence for population stratification. For constrained LDSC correlation analysis, we constrained in addition the intercept for genetic covariance estimations to zero. i.e. in the presence of no sample overlap.

Polygenic scoring analysis

Utilising a series of clinical 'training' samples^14^, polygenic scores were generated in the population-based ALSPAC cohort as a 'target' set. Common autosomal signals observed in clinical samples (with MAF>0.01 in ALSPAC) were clumped (LD-r^2^>0.25, ±500 kb) consistent with current guidelines^15^ using PLINK^16^, excluding duplicate SNPs. For schizophrenia signals, derived from highly powerful but heterogeneous PGC-SCZ2 samples^7^, we selected markers with very high imputation accuracy (INFO>0.9, as observed in PGC-SCZ2) and excluded variants within the MHC-region (chromosome 6: 26 to 33Mb), following protocols of the PGC^7^. Excluding MHC-variants avoids an overrepresentation of schizophrenia-related signals due to extended LD. Polygenic scores were constructed using PGC-SCZ1, PGC-SCZ2i and PGC-SCZ2 summary statistics (Table 1). For ASD signals in PGC-ASD, we selected markers with INFO>0.8, given the ethnically homogeneous sample and the robust trio design^17^. Using a range of *P*-value thresholds (0.001<*P_T_*≤ 1), polygenic risk scores for ASD and schizophrenia were generated in ALSPAC using the best imputed common genotypes with respect to a 1000 genomes template (PhaseI_v3, INFO>0.8). Rank-transformed SCDC scores were regressed on Z-standardised PGS using OLS regression (Rv3.2.2), and the proportion of phenotypic variance explained by each PGS predictor reported as Adjusted-R^2^.

Genome-wide Complex Trait Analysis

Genome-wide Complex Trait Analysis (GCTA) was utilised to estimate the proportion of additive phenotypic variation in rank-transformed SCDC scores jointly explained by SNPs on a genotyping chip (SNP-h^2^) using genetic-relationship-matrix residual maximum likelihood (GREML)^18^. In addition, bivariate GCTA^19^ was carried out to estimate the extent (i.e. r_g_) to which repeatedly assessed SCDC scores share the same genetic factors. GREML analyses are similar to previously published work^3^ and were carried out for comparison only.

Attrition within ALSPAC

To study attrition patterns within ALSPAC, analysis was restricted to participants, who were alive at one year of age and had information on genome-wide data available (N=7,758). Dichotomic SCDC-missingness was defined as availability of mother-reported scores at 8, 11, 14 and 17 years of age. A logistic regression framework (Rv3.2.2) was utilised to estimate odds ratios and their standard errors per one-standard-deviation increase in polygenic scores (see Methods). McFadden's pseudo-R^2^ values for logistic regression were calculated, analogous to the ordinary least square regression R^2^. Analyses were furthermore adjusted for maternal educational levels (low: Certificate of Secondary Education or vocational; high: O-levels, A-levels, or undergraduate degree), a potential correlate for non-participation^20^.

**Supplementary references**

1 Delaneau O, Zagury J-F, Marchini J. Improved whole-chromosome phasing for disease and population genetic studies. *Nat Methods* 2013; **10**: 5–6.

2 Marchini J, Howie B, Myers S, McVean G, Donnelly P. A new multipoint method for genome-wide association studies by imputation of genotypes. *Nat Genet* 2007; **39**: 906–913.

3 St Pourcain B, Skuse DH, Mandy WP, Wang K, Hakonarson H, Timpson NJ *et al.* Variability in the common genetic architecture of social-communication spectrum phenotypes during childhood and adolescence. *Mol Autism* 2014; **5**: 18.

4 Hollegaard MV, Grove J, Grauholm J, Kreiner-Møller E, Bønnelykke K, Nørgaard M *et al.* Robustness of genome-wide scanning using archived dried blood spot samples as a DNA source. *BMC Genet* 2011; **12**: 58.

5 Børglum AD, Demontis D, Grove J, Pallesen J, Hollegaard MV, Pedersen CB *et al.* Genome-wide study of association and interaction with maternal cytomegalovirus infection suggests new schizophrenia loci. *Mol Psychiatry* 2014; **19**: 325–333.

6 *The ICD-10 Classification of Mental and Behavioural Disorders: Clinical Descriptions and Diagnostic Guidelines*. 1 edition. World Health Organization: Geneva, 1992.

7 Schizophrenia Working Group of the Psychiatric Genomics Consortium. Biological insights from 108 schizophrenia-associated genetic loci. *Nature* 2014; **511**: 421–427.

8 Williams E, Thomas K, Sidebotham H, Emond A. Prevalence and characteristics of autistic spectrum disorders in the ALSPAC cohort. *Dev Med Child Neurol* 2008; **50**: 672–677.

9 Ripke S, Wray NR, Lewis CM, Hamilton SP, Weissman MM, Breen G *et al.* A mega-analysis of genome-wide association studies for major depressive disorder. *Mol Psychiatry* 2013; **18**: 497–511.

10 Psychiatric GWAS Consortium Bipolar Disorder Working Group. Large-scale genome-wide association analysis of bipolar disorder identifies a new susceptibility locus near ODZ4. *Nat Genet* 2011; **43**: 977–983.

11 Bulik-Sullivan BK, Loh P-R, Finucane HK, Ripke S, Yang J, Schizophrenia Working Group of the Psychiatric Genomics Consortium *et al.* LD Score regression distinguishes confounding from polygenicity in genome-wide association studies. *Nat Genet* 2015; **47**: 291–295.

12 Bulik-Sullivan B, Finucane HK, Anttila V, Gusev A, Day FR, Loh P-R *et al.* An atlas of genetic correlations across human diseases and traits. *Nat Genet* 2015; **47**: 1236–1241.

13 International HapMap 3 Consortium. Integrating common and rare genetic variation in diverse human populations. *Nature* 2010; **467**: 52–58.

14 Purcell SM, Wray NR, Stone JL, Visscher PM, O’Donovan MC, Sullivan PF *et al.* Common polygenic variation contributes to risk of schizophrenia and bipolar disorder. *Nature* 2009; **460**: 748–52.

15 Wray NR, Lee SH, Mehta D, Vinkhuyzen AAE, Dudbridge F, Middeldorp CM. Research Review: Polygenic methods and their application to psychiatric traits. *J Child Psychol Psychiatry* 2014; **55**: 1068–1087.

16 Purcell S, Neale B, Todd-Brown K, Thomas L, Ferreira MAR, Bender D *et al.* PLINK: A Tool Set for Whole-Genome Association and Population-Based Linkage Analyses. *Am J Hum Genet* 2007; **81**: 559–575.

17 Cross-Disorder Group of the Psychiatric Genomics Consortium]. Identification of risk loci with shared effects on five major psychiatric disorders: a genome-wide analysis. *The Lancet* 2013; **381**: 1371–1379.

18 Yang J, Benyamin B, McEvoy BP, Gordon S, Henders AK, Nyholt DR *et al.* Common SNPs explain a large proportion of the heritability for human height. *Nat Genet* 2010; **42**: 565–569.

19 Lee SH, Yang J, Goddard ME, Visscher PM, Wray NR. Estimation of pleiotropy between complex diseases using single-nucleotide polymorphism-derived genomic relationships and restricted maximum likelihood. *Bioinformatics* 2012; **28**: 2540–2542.

20 Martin J, Tilling K, Hubbard L, Stergiakouli E, Thapar A, Smith GD *et al.* Association of Genetic Risk for Schizophrenia With Nonparticipation Over Time in a Population-Based Cohort Study. *Am J Epidemiol* 2016; : kww009.

**iPSYCH‐SSI‐Broad Autism Group**

Thomas D. Als, Marie Baekvad‐Hansen, Richard Belliveau, Anders D. Børglum, Mark J. Daly, Ditte Demontis, Ashley Dumont, Jacqueline Goldstein, Jonas Grauholm, Jakob Grove, Christine S. Hansen, Thomas F. Hansen, Mads V. Hollegaard, Daniel Howrigan, David M. Hougaard, Francesco Lescai, Julian Maller, Joanna Martin, Manuel Mattheisen, Jennifer Moran, Preben Bo Mortensen, Ole Mors, Benjamin N. Neale, Merete Nordentoft, Bent Nørgaard-Pedersen, Timothy Poterba, Jesper Poulsen, Elise B. Robinson, Stephan Ripke, Christine Stevens, Raymond Walters, and Thomas Werge

**Web resources**

1000genomes: http://www.1000genomes.org/

ALSPAC: http://www.bris.ac.uk/alspac/researchers/data--‐access/data--‐dictionary

HapMap3: <https://www.sanger.ac.uk/resources/downloads/human/hapmap3.html>

ASHA : http://www.asha.org/

NIH: http://www.nimh.nih.gov/research-priorities/rdoc/index.shtml

LDSC: http://www.github.com/bulik/ldsc

PGC: <http://www.med.unc.edu/pgc/>

PLINK2: http://pngu.mgh.harvard.edu/~purcell/plink/plink2.shtml

R: [https://cran.r-project.org/](http://cran.r-project.org/)

SNPTEST: <https://mathgen.stats.ox.ac.uk/genetics_software/snptest/snptest.html>

**Supplementary tables**

**Supplementary Table 1:** Descriptives of SCDC scores

| Trait | Age(years)[range] | Male/Female | Mean(SD) [range] | N |
| --- | --- | --- | --- | --- |
| 8 | 7.7(0.14)[7.5;9.3] | 2843/2710 | 2(3.72)[0;24] | 5553 |
| 11 | 10.7(0.13)[10.5;13.8] | 2752/2710 | 1(3.51)[0;24] | 5462 |
| 14 | 13.9(0.15)[13.7;16.1] | 2529/2531 | 1(3.59)[0;24] | 5060 |
| 17 | 16.8(0.36)[16.5;18.3] | 2024/2151 | 1(3.79)[0;24] | 4175 |

ALSPAC - Avon Longitudinal study of Parents and Children; SCDC - Social-Communication Disorder Checklist assessed in ALSPAC youth

**Supplementary Table 2:** Phenotypic correlations of SCDC scores

| **Age in years** | | | | |
| --- | --- | --- | --- | --- |
|  | **8** | **11** | **14** | **17** |
| **8** | 1.00 | 0.61 | 0.50 | 0.38 |
| **11** | 0.57 | 1.00 | 0.56 | 0.41 |
| **14** | 0.49 | 0.57 | 1.00 | 0.51 |
| **17** | 0.39 | 0.45 | 0.56 | 1.00 |

ALSPAC - Avon Longitudinal study of Parents and Children; SCDC - Social-Communication Disorder Checklist assessed in ALSPAC youth

Lower triangle: Spearman’s rank correlation using pairwise complete observations; Upper triangle: Pearson product moment correlation using rank-transformed scores adjusted for age, sex and the two most significant ancestry-informative principal components; Estimates correspond closely to previously published correlations ^3^

Note, that bivariate correlation patterns only approximate multivariate correlation patterns and may not accurately reflect phenotypic correlations over time.

**Supplementary Table 3:** Genetic correlations of SCDC scores

| **GCTA-r_g_ (SE) and *P*** | | | | |
| --- | --- | --- | --- | --- |
| **Age** | **8** | **11** | **14** | **17** |
| **8** | - | 1x10^-4^ | 0.02 | 9x10^-4^ |
| **11** | 0.93(0.14) | - | 0.02 | 0.02 |
| **14** | 0.78(0.31) | 0.82(0.24) | - | 2x10^-7^ |
| **17** | 0.50(0.14) | 0.38(0.16) | 0.95(0.34) | - |

ALSPAC - Avon Longitudinal study of Parents and Children; GCTA - Genome-wide Complex Trait Analysis; GCTA-r_g_ - Bivariate GCTA genetic correlation; SCDC - Social-Communication Disorder Checklist assessed in ALSPAC youth

Findings correspond closely to previously published estimates^3^

**Supplementary Table 4:** Genetic correlations between psychiatric disorder and SCDC scores

| ***ASD*** | | | |
| --- | --- | --- | --- |
| **Clinical sample** | **SCDC**  **(ALSPAC)** | **r_g_ (SE), *P***  **(intercept constraints)** | **r_g_ (SE), *P***  **(unconstrained intercepts)** |
| PGC-ASD^a^ | 8 y | **0.34(0.15), *P*=0.027** | 0.18(0.17), *P*=0.27 |
|  | 11 y | 0.16(0.15), *P*=0.29 | 0.03(0.17), *P*=0.88 |
|  | 14 y | 0.21(0.27), *P*=0.43 | 0.15(0.2), *P*=0.46 |
|  | 17 y | 0.01(0.12), *P*=0.94 | -0.06(0.15), *P*=0.68 |
| iPSYCH-ASD | 8 y | **0.35(0.13), *P*=0.008** | **0.34(0.15), *P*=0.021** |
|  | 11 y | **0.33(0.14), *P*=0.018** | 0.2(0.19), *P*=0.28 |
|  | 14 y | 0.28(0.21), *P*=0.173 | 0.21(0.19), *P*=0.28 |
|  | 17 y | 0.02(0.10), *P*=0.81 | -0.08(0.18), *P*=0.67 |
| ***Schizophrenia*** | | | |
| **Clinical sample** | **SCDC**  **(ALSPAC)** | **r_g_ (SE), *P***  **(intercept constraints)** | **r_g_ (SE), *P***  **(unconstrained intercepts)** |
| PGC-SCZ1 | 8 y | **0.20(0.08), *P*=0.010** | 0.18(0.10), *P*=0.06 |
|  | 11 y | **0.18(0.09), *P*=0.044** | 0.05(0.11), *P*=0.66 |
|  | 14 y | 0.20(0.14), *P*=0.16 | 0.09(0.1), *P*=0.41 |
|  | 17 y | **0.24(0.08), *P*=0.004** | 0.14(0.13), *P*=0.28 |
| PGC-SCZ2i | 8 y | 0.08(0.06), *P*=0.16 | 0.06(0.09), *P*=0.53 |
|  | 11 y | 0.10(0.07), *P*=0.16 | 0.04(0.09), *P*=0.65 |
|  | 14 y | 0.18(0.12), *P*=0.12 | 0.03(0.1), *P*=0.73 |
|  | 17 y | **0.15(0.06), *P*=0.011** | 0.12(0.11), *P*=0.28 |
| PGC-SCZ2 | 8 y | **0.12(0.06), *P*=0.04** | 0.11(0.08), *P*=0.20 |
|  | 11 y | 0.13(0.07), *P*=0.065 | 0.04(0.08), *P*=0.66 |
|  | 14 y | 0.19(0.13), *P*=0.14 | 0.08(0.1), *P*=0.43 |
|  | 17 y | **0.18(0.06), *P*=0.003** | 0.17(0.14), *P*=0.22 |
| PGC-SCZ2-Eur | 8 y | **0.12(0.06), *P*=0.034** | 0.11(0.09), *P*=0.21 |
|  | 11 y | 0.12(0.07), *P*=0.061 | 0.02(0.08), *P*=0.84 |
|  | 14 y | 0.18(0.11), *P*=0.12 | 0.06(0.1), *P*=0.57 |
|  | 17 y | **0.18(0.06), *P*=0.004** | 0.15(0.14), *P*=0.26 |
| ***Other adult-onset psychiatric disorders^b^*** | | | |
|  | **SCDC**  **(ALSPAC)** | **r_g_ (SE), *P***  **(intercept constraints)** | **r_g_ (SE), *P***  **(unconstrained intercepts)** |
| PGC-MDD | 17 y | -0.05(0.11), *P*=0.65 | 0.20(0.22), *P*=0.37 |
| PGC-BIP | 17 y | 0.04(0.08), *P*=0.62 | -0.02(0.16), *P*=0.90 |

Genetic correlations (r_g_) were estimated using LD-score correlation with and without intercept constraints^11^; a - PGC-ASD is by design free of population stratification; b - Sensitivity analysis; *P-*values < 0.05 are indicated in bold

ALSPAC - Avon Longitudinal Study of Parents and Children; ASD - Autism Spectrum Disorders; BIP - Bipolar disorder; iPSYCH-ASD - iPSYCH-SSI-BROAD Autism project; MDD - Major depressive disorder; PGC - Psychiatric Genomics Consortium; PGC-ASD - ASD collection of the PGC; PGC-BIP - PGC mega-analysis of BIP; PGC-MDD - PGC mega-analysis of MDD; PGC-SCZ1 - First PGC mega-analysis of SCZ; PGC-SCZ2i - PGC-SCZ2 samples not analysed within PGC-SCZ1; PGC-SCZ2 - Second PGC mega-analysis of SCZ (PGC-SCZ1 + PGC-SCZ2i); PGC-SCZ2-Eur - PGC-SCZ2 samples of European ancestry only (exclusion of 1,836 cases and 3,383 controls from East Asia)^16^; SCDC - Social-Communication Disorder Checklist; SCZ - Schizophrenia; y - years

**Supplementary Table 5:** Genetic correlations between schizophrenia and other psychiatric disorders

| ***PGC-ASD^a^*** | | |
| --- | --- | --- |
| **Schizophrenia sample** | **r_g_ (SE), *P***  **(intercept constraints)** | **r_g_ (SE), *P***  **(unconstrained intercepts)** |
| PGC-SCZ1 | 0.20(0.04), *P*=5.5x10^-7^ | 0.19(0.06), *P*=0.0055 |
| PGC-SCZ2i | 0.23(0.05), *P*=2.5x10^-6^ | 0.16(0.06), *P*=0.0048 |
| PGC-SCZ2 | 0.19(0.04), *P*=1.7x10^-5b^ | 0.20(0.05), *P*=0.00011^b^ |
| ***PGC-BIP*** | | |
| **Schizophrenia sample** | **r_g_ (SE), *P***  **(intercept constraints)** | **r_g_ (SE), *P***  **(unconstrained intercepts)** |
| PGC-SCZ1 | - | 0.78(0.04), *P*=1.7x10^-92^ |
| PGC-SCZ2i | - | 0.80(0.04), *P*=1.3x10^-75^ |
| PGC-SCZ2 | - | 0.76(0.04), *P*=6.5x10^-70^ |
| ***PGC-MDD*** | | |
| **Schizophrenia sample** | **r_g_ (SE), *P***  **(intercept constraints)** | **r_g_ (SE), *P***  **(unconstrained intercepts)** |
| PGC-SCZ1 | - | 0.51(0.07), *P*=1.2x10^-13^ |
| PGC-SCZ2i | - | 0.50(0.07), *P*=6.2 x10^-12^ |
| PGC-SCZ2 | - | 0.49(0.08), *P*=4.3 x10^-11^ |

Genetic correlations (r_g_) were estimated using LD-score correlation with and without intercept constraints^12^; a - PGC-ASD is by design free of population stratification; b - Estimates correspond to previously reported findings using Genome-wide Complex Trait Analysis (r_g_(SE)=0.16(0.06), *P*=0.0071)^17^

ASD - Autism Spectrum Disorders; PGC - Psychiatric Genomics Consortium; BIP - Bipolar disorder; MDD - Major depressive disorder; PGC - Psychiatric Genomics Consortium; PGC-ASD - ASD collection of the PGC; PGC-BIP - PGC mega-analysis of BIP; PGC-MDD - PGC mega-analysis of MDD; PGC-SCZ1 - First PGC mega-analysis of SCZ; PGC-SCZ2i - PGC-SCZ2 samples not analysed within PGC-SCZ1; PGC-SCZ2 - Second PGC mega-analysis of SCZ (PGC-SCZ1 + PGC-SCZ2i); SCZ - Schizophrenia

Analyses correspond closely to previously published PGC results^12^

**Supplementary Table 6:** Association of ASD and schizophrenia risk scores with SCDC scores

|  |  | **ASD-PGS (PGC-ASD)** | | | | | | **Schizophrenia-PGS (PGC-SCZ2)** | | | | | |
| --- | --- | --- | --- | --- | --- | --- | --- | --- | --- | --- | --- | --- | --- |
| **SCDC** | **PGS bin** | **Unadjusted** | | | **Adjusted** | | | **Unadjusted** | | | **Adjusted** | | |
|  |  | **Beta (SE)** | **OLS *P*** | **Adj-R^2^ (%)** | **Beta (SE)** | **OLS *P*** | **Adj-R^2^ (%)** | **Beta (SE)** | **OLS *P*** | **Adj-R^2^ (%)** | **Beta (SE)** | **OLS *P*** | **Adj-R^2^ (%)** |
| 8 y | P_T_<0.001 | **0.028(0.01)** | **0.033** | **0.06** | **0.029(0.01)** | **0.030** | **0.09** | 0.022(0.01) | 0.093 | - | 0.023(0.01) | 0.082 | na |
|  | P_T_<0.01 | **0.032(0.01)** | **0.016** | **0.09** | **0.033(0.01)** | **0.012** | **0.11** | **0.026(0.01)** | **0.05** | **0.05** | **0.028(0.01)** | **0.039** | **0.08** |
|  | P_T_<0.05 | **0.037(0.01)** | **0.0055** | **0.12** | **0.038(0.01)** | **0.0046** | **0.14** | **0.029(0.01)** | **0.034** | **0.06** | **0.03(0.01)** | **0.028** | **0.09** |
|  | P_T_<0.1 | **0.036(0.01)** | **0.0076** | **0.11** | **0.036(0.01)** | **0.0076** | **0.13** | **0.034(0.01)** | **0.011** | **0.10** | **0.034(0.01)** | **0.011** | **0.12** |
|  | P_T_<0.3 | **0.038(0.01)** | **0.0042** | **0.13** | **0.038(0.01)** | **0.0043** | **0.15** | **0.034(0.01)** | **0.011** | **0.10** | **0.034(0.01)** | **0.012** | **0.11** |
|  | P_T_<0.5 | **0.036(0.01)** | **0.0062** | **0.12** | **0.036(0.01)** | **0.0065** | **0.13** | **0.035(0.01)** | **0.009** | **0.10** | **0.035(0.01)** | **0.0094** | **0.12** |
|  | P_T_<0.7 | **0.037(0.01)** | **0.0056** | **0.12** | **0.037(0.01)** | **0.0059** | **0.14** | **0.036(0.01)** | **0.0084** | **0.11** | **0.035(0.01)** | **0.0087** | **0.12** |
|  | P_T_<0.9 | **0.037(0.01)** | **0.0050** | **0.12** | **0.037(0.01)** | **0.0052** | **0.14** | **0.036(0.01)** | **0.0072** | **0.11** | **0.036(0.01)** | **0.0075** | **0.13** |
|  | P_T_<1 | **0.037(0.01)** | **0.0049** | **0.12** | **0.037(0.01)** | **0.0051** | **0.14** | **0.036(0.01)** | **0.0073** | **0.11** | **0.036(0.01)** | **0.0076** | **0.13** |
| 11 y | P_T_<0.001 | 0.012(0.01) | 0.39 | - | 0.012(0.01) | 0.37 | na | 0.016(0.01) | 0.22 | - | 0.017(0.01) | 0.22 | na |
|  | P_T_<0.01 | 0.015(0.01) | 0.26 | - | 0.016(0.01) | 0.24 | na | 0.021(0.01) | 0.13 | - | 0.021(0.01) | 0.12 | na |
|  | P_T_<0.05 | 0.016(0.01) | 0.24 | - | 0.016(0.01) | 0.23 | na | **0.029(0.01)** | **0.035** | **0.06** | **0.029(0.01)** | **0.033** | **0.08** |
|  | P_T_<0.1 | 0.016(0.01) | 0.23 | - | 0.016(0.01) | 0.24 | na | **0.03(0.01)** | **0.027** | **0.07** | **0.03(0.01)** | **0.027** | **0.09** |
|  | P_T_<0.3 | 0.011(0.01) | 0.41 | - | 0.011(0.01) | 0.41 | na | **0.033(0.01)** | **0.016** | **0.09** | **0.033(0.01)** | **0.016** | **0.11** |
|  | P_T_<0.5 | 0.011(0.01) | 0.42 | - | 0.011(0.01) | 0.42 | na | **0.034(0.01)** | **0.012** | **0.10** | **0.034(0.01)** | **0.012** | **0.12** |
|  | P_T_<0.7 | 0.01(0.01) | 0.45 | - | 0.01(0.01) | 0.46 | na | **0.035(0.01)** | **0.01** | **0.10** | **0.035(0.01)** | **0.01** | **0.12** |
|  | P_T_<0.9 | 0.01(0.01) | 0.47 | - | 0.01(0.01) | 0.48 | na | **0.036(0.01)** | **0.0086** | **0.11** | **0.036(0.01)** | **0.0087** | **0.13** |
|  | P_T_<1 | 0.01(0.01) | 0.46 | - | 0.01(0.01) | 0.47 | na | **0.036(0.01)** | **0.0088** | **0.11** | **0.035(0.01)** | **0.0089** | **0.13** |
| 14 y | P_T_<0.001 | 0.024(0.01) | 0.08 | - | 0.024(0.01) | 0.079 | na | 0.003(0.01) | 0.84 | - | 0.003(0.01) | 0.81 | na |
|  | P_T_<0.01 | 0.005(0.01) | 0.72 | - | 0.005(0.01) | 0.7 | na | 0.017(0.01) | 0.22 | - | 0.017(0.01) | 0.22 | na |
|  | P_T_<0.05 | 0(0.01) | 0.97 | - | 0.001(0.01) | 0.96 | na | 0.021(0.01) | 0.14 | - | 0.021(0.01) | 0.14 | na |
|  | P_T_<0.1 | 0.007(0.01) | 0.64 | - | 0.006(0.01) | 0.67 | na | **0.03(0.01)** | **0.031** | **0.07** | **0.03(0.01)** | **0.031** | **0.09** |
|  | P_T_<0.3 | 0.001(0.01) | 0.96 | - | 0(0.01) | 0.99 | na | **0.034(0.01)** | **0.015** | **0.10** | **0.034(0.01)** | **0.016** | **0.12** |
|  | P_T_<0.5 | -0.002(0.01) | 0.88 | - | -0.003(0.01) | 0.86 | na | **0.033(0.01)** | **0.019** | **0.09** | **0.033(0.01)** | **0.019** | **0.11** |
|  | P_T_<0.7 | -0.001(0.01) | 0.92 | - | -0.002(0.01) | 0.89 | na | **0.033(0.01)** | **0.02** | **0.09** | **0.033(0.01)** | **0.02** | **0.11** |
|  | P_T_<0.9 | -0.002(0.01) | 0.91 | - | -0.002(0.01) | 0.88 | na | **0.033(0.01)** | **0.019** | **0.09** | **0.033(0.01)** | **0.019** | **0.11** |
|  | P_T_<1 | -0.002(0.01) | 0.91 | - | -0.002(0.01) | 0.88 | na | **0.033(0.01)** | **0.02** | **0.09** | **0.033(0.01)** | **0.02** | **0.11** |
| 17 y | P_T_<0.001 | 0.001(0.02) | 0.95 | - | 0.002(0.02) | 0.89 | na | **0.04(0.02)** | **0.0089** | **0.14** | **0.04(0.02)** | **0.0088** | **0.16** |
|  | P_T_<0.01 | 0.01(0.02) | 0.51 | - | 0.012(0.02) | 0.43 | na | **0.061(0.02)** | **0.000086** | **0.34** | **0.061(0.02)** | **0.000079** | **0.37** |
|  | P_T_<0.05 | 0.009(0.02) | 0.57 | - | 0.01(0.02) | 0.53 | na | **0.062(0.02)** | **0.000054** | **0.37** | **0.062(0.02)** | **0.000052** | **0.39** |
|  | P_T_<0.1 | 0.009(0.02) | 0.56 | - | 0.009(0.02) | 0.56 | na | **0.062(0.02)** | **0.000066** | **0.36** | **0.062(0.02)** | **0.000066** | **0.38** |
|  | P_T_<0.3 | 0(0.02) | 0.99 | - | 0(0.02) | 0.99 | na | **0.066(0.02)** | **0.000022** | **0.41** | **0.066(0.02)** | **0.000022** | **0.43** |
|  | P_T_<0.5 | 0(0.02) | 1.00 | - | 0(0.02) | 1 | na | **0.068(0.02)** | **0.000012** | **0.43** | **0.068(0.02)** | **0.000012** | **0.46** |
|  | P_T_<0.7 | -0.001(0.02) | 0.93 | - | -0.001(0.02) | 0.93 | na | **0.067(0.02)** | **0.000016** | **0.42** | **0.067(0.02)** | **0.000016** | **0.45** |
|  | P_T_<0.9 | 0(0.02) | 0.98 | - | 0(0.02) | 0.98 | na | **0.066(0.02)** | **0.000018** | **0.42** | **0.066(0.02)** | **0.000018** | **0.44** |
|  | P_T_<1 | 0(0.02) | 0.98 | - | 0(0.02) | 0.99 | na | **0.066(0.02)** | **0.000018** | **0.42** | **0.066(0.02)** | **0.000018** | **0.44** |

Polygenic scores for risk-increasing alleles (PGS) were constructed in ALSPAC then Z-standardised. Rank-transformed SCDC scores (at 8, 11, 14 and 17 years) were regressed on ASD-PGS and schizophrenia-PGS using OLS, and are shown by PGS bin; Adjusted-R^2^ (Adj-R^2^) is reported for *P*<0.05 ( indicated in bold); Unadjusted/Adjusted - OLS regression with ASD-PGS and schizophrenia-PGS adjusted for each other, and unadjusted otherwise; ALSPAC - Avon Longitudinal Study of Parents and Children; ASD - Autism Spectrum Disorders; na - Not available; PGC - Psychiatric Genomics Consortium ; PGC-ASD - ASD collection of the PGC; *P*_T_ - PGS threshold; OLS - Ordinary Least Square regression; PGC-SCZ2 - Second PGC mega-analysis of SCZ; SCDC - Social-Communication Disorder Checklist; SCZ - Schizophrenia; y - years

**Supplementary Table 7:** Association of schizophrenia risk scores with SCDC scores (PGC-SCZ2 subsamples)

| **SCDC** | **PGS bin** | **Schizophrenia-PGS (SCZ-PGC1)** | | | **Schizophrenia-PGS (SCZ-PGC2i)** | | |
| --- | --- | --- | --- | --- | --- | --- | --- |
|  |  | **Beta (SE)** | **OLS *P*** | **Adj-R^2^ (%)** | **Beta (SE)** | **OLS *P*** | **Adj-R^2^ (%)** |
| 8 y | P_T_<0.001 | 0.023(0.01) | 0.084 | na | 0.021(0.01) | 0.12 | na |
|  | P_T_<0.01 | **0.028(0.01)** | **0.037** | **0.06** | 0.004(0.01) | 0.77 | na |
|  | P_T_<0.05 | **0.035(0.01)** | **0.009** | **0.1** | 0.011(0.01) | 0.41 | na |
|  | P_T_<0.1 | **0.027(0.01)** | **0.047** | **0.05** | 0.014(0.01) | 0.31 | na |
|  | P_T_<0.3 | **0.027(0.01)** | **0.041** | **0.06** | 0.021(0.01) | 0.12 | na |
|  | P_T_<0.5 | **0.029(0.01)** | **0.033** | **0.06** | 0.02(0.01) | 0.13 | na |
|  | P_T_<0.7 | **0.028(0.01)** | **0.037** | **0.06** | 0.02(0.01) | 0.13 | na |
|  | P_T_<0.9 | **0.028(0.01)** | **0.034** | **0.06** | 0.019(0.01) | 0.15 | na |
|  | P_T_<1 | **0.029(0.01)** | **0.033** | **0.06** | 0.019(0.01) | 0.15 | na |
| 11 y | P_T_<0.001 | 0.01(0.01) | 0.47 | na | 0.007(0.01) | 0.58 | na |
|  | P_T_<0.01 | **0.037(0.01)** | **0.007** | **0.11** | -0.005(0.01) | 0.71 | na |
|  | P_T_<0.05 | **0.034(0.01)** | **0.012** | **0.1** | 0.018(0.01) | 0.19 | na |
|  | P_T_<0.1 | **0.039(0.01)** | **0.0041** | **0.13** | 0.016(0.01) | 0.24 | na |
|  | P_T_<0.3 | **0.037(0.01)** | **0.0078** | **0.11** | 0.022(0.01) | 0.11 | na |
|  | P_T_<0.5 | **0.035(0.01)** | **0.011** | **0.1** | 0.022(0.01) | 0.1 | na |
|  | P_T_<0.7 | **0.035(0.01)** | **0.0099** | **0.1** | 0.022(0.01) | 0.1 | na |
|  | P_T_<0.9 | **0.035(0.01)** | **0.011** | **0.1** | 0.022(0.01) | 0.1 | na |
|  | P_T_<1 | **0.035(0.01)** | **0.011** | **0.1** | 0.022(0.01) | 0.1 | na |
| 14 y | P_T_<0.001 | -0.008(0.01) | 0.56 | na | 0.017(0.01) | 0.24 | na |
|  | P_T_<0.01 | 0.008(0.01) | 0.58 | na | -0.006(0.01) | 0.69 | na |
|  | P_T_<0.05 | 0.023(0.01) | 0.11 | na | 0.018(0.01) | 0.2 | na |
|  | P_T_<0.1 | 0.022(0.01) | 0.12 | na | 0.017(0.01) | 0.21 | na |
|  | P_T_<0.3 | 0.017(0.01) | 0.24 | na | **0.028(0.01)** | **0.046** | **0.06** |
|  | P_T_<0.5 | 0.013(0.01) | 0.37 | na | 0.027(0.01) | 0.056 | na |
|  | P_T_<0.7 | 0.013(0.01) | 0.34 | na | 0.026(0.01) | 0.061 | na |
|  | P_T_<0.9 | 0.014(0.01) | 0.33 | na | 0.026(0.01) | 0.061 | na |
|  | P_T_<1 | 0.014(0.01) | 0.33 | na | 0.026(0.01) | 0.061 | na |
| 17 y | P_T_<0.001 | **0.033(0.02)** | **0.036** | **0.08** | **0.034(0.02)** | **0.028** | **0.09** |
|  | P_T_<0.01 | **0.046(0.02)** | **0.0032** | **0.18** | **0.034(0.02)** | **0.027** | **0.09** |
|  | P_T_<0.05 | **0.052(0.02)** | **0.00083** | **0.24** | **0.035(0.02)** | **0.022** | **0.1** |
|  | P_T_<0.1 | **0.05(0.02)** | **0.0012** | **0.23** | **0.032(0.02)** | **0.039** | **0.08** |
|  | P_T_<0.3 | **0.054(0.02)** | **0.00058** | **0.26** | **0.042(0.02)** | **0.0058** | **0.16** |
|  | P_T_<0.5 | **0.052(0.02)** | **0.00083** | **0.24** | **0.044(0.02)** | **0.0038** | **0.18** |
|  | P_T_<0.7 | **0.053(0.02)** | **0.00062** | **0.26** | **0.046(0.02)** | **0.0028** | **0.19** |
|  | P_T_<0.9 | **0.052(0.02)** | **0.00073** | **0.25** | **0.046(0.02)** | **0.0029** | **0.19** |
|  | P_T_<1 | **0.052(0.02)** | **0.00072** | **0.25** | **0.046(0.02)** | **0.0028** | **0.19** |

Polygenic scores for risk-increasing alleles (PGS) were constructed in ALSPAC based on two independent schizophrenia samples (PGC-SCZ1 and PGC-SCZ2i) as a training set, and then Z-standardised. Rank transformed SCDC scores (at 8, 11, 14 and 17 years) in ALSPAC were regressed on schizophrenia-PGS using OLS, and are shown by PGS bin; Adjusted-R^2^ (Adj-R^2^) is reported for *P*<0.05 ( indicated in bold);

ALSPAC - Avon Longitudinal Study of Parents and Children; na - Not available; PGC - Psychiatric Genomics Consortium ; P_T_ - PGS threshold; OLS - Ordinary Least Square regression; PGC-SCZ1- First PGC mega-analysis of SCZ; PGC-SCZ2i - PGC-SCZ2 samples not analysed within PGC-SCZ1; SCDC - Social-Communication Disorder Checklist; SCZ - Schizophrenia; y - years

**Supplementary Table 8:** Association of ASD and schizophrenia risk scores with SCDC scores (exclusion of ALSPAC ASD cases)

| **SCDC** | **PGS bin** | **ASD-PGS (PGC-ASD)** | | | **Schizophrenia-PGS (PGC-SCZ2)** | | |
| --- | --- | --- | --- | --- | --- | --- | --- |
|  |  | **Beta (SE)** | ***P*** | **Adj-R^2^ (%)** | **Beta (SE)** | ***P*** | **Adj-R^2^ (%)** |
| 8 y | P_T_<0.001 | **0.028(0.01)** | **0.034** | **0.06** | 0.02(0.01) | 0.14 | na |
|  | P_T_<0.01 | **0.027(0.01)** | **0.038** | **0.06** | 0.022(0.01) | 0.10 | na |
|  | P_T_<0.05 | **0.03(0.01)** | **0.024** | **0.07** | 0.025(0.01) | 0.058 | na |
|  | P_T_<0.1 | **0.029(0.01)** | **0.027** | **0.07** | **0.031(0.01)** | **0.022** | **0.08** |
|  | P_T_<0.3 | **0.032(0.01)** | **0.017** | **0.09** | **0.03(0.01)** | **0.024** | **0.07** |
|  | P_T_<0.5 | **0.03(0.01)** | **0.023** | **0.08** | **0.032(0.01)** | **0.018** | **0.08** |
|  | P_T_<0.7 | **0.03(0.01)** | **0.022** | **0.08** | **0.032(0.01)** | **0.017** | **0.08** |
|  | P_T_<0.9 | **0.031(0.01)** | **0.020** | **0.08** | **0.032(0.01)** | **0.015** | **0.09** |
|  | P_T_<1 | **0.031(0.01)** | **0.020** | **0.08** | **0.032(0.01)** | **0.015** | **0.09** |
| 11 y | P_T_<0.001 | 0.01(0.01) | 0.46 | na | 0.015(0.01) | 0.26 | na |
|  | P_T_<0.01 | 0.011(0.01) | 0.43 | na | 0.018(0.01) | 0.19 | na |
|  | P_T_<0.05 | 0.01(0.01) | 0.48 | na | **0.027(0.01)** | **0.047** | **0.05** |
|  | P_T_<0.1 | 0.011(0.01) | 0.42 | na | **0.028(0.01)** | **0.042** | **0.06** |
|  | P_T_<0.3 | 0.007(0.01) | 0.63 | na | **0.03(0.01)** | **0.029** | **0.07** |
|  | P_T_<0.5 | 0.006(0.01) | 0.65 | na | **0.031(0.01)** | **0.020** | **0.08** |
|  | P_T_<0.7 | 0.005(0.01) | 0.70 | na | **0.032(0.01)** | **0.018** | **0.08** |
|  | P_T_<0.9 | 0.005(0.01) | 0.73 | na | **0.032(0.01)** | **0.016** | **0.09** |
|  | P_T_<1 | 0.005(0.01) | 0.72 | na | **0.032(0.01)** | **0.016** | **0.09** |
| 14 y | P_T_<0.001 | 0.024(0.01) | 0.086 | na | 0.002(0.01) | 0.89 | na |
|  | P_T_<0.01 | 0.001(0.01) | 0.96 | na | 0.015(0.01) | 0.30 | na |
|  | P_T_<0.05 | -0.004(0.01) | 0.77 | na | 0.018(0.01) | 0.20 | na |
|  | P_T_<0.1 | 0.002(0.01) | 0.89 | na | **0.028(0.01)** | **0.049** | **0.06** |
|  | P_T_<0.3 | -0.004(0.01) | 0.78 | na | **0.031(0.01)** | **0.027** | **0.08** |
|  | P_T_<0.5 | -0.006(0.01) | 0.64 | na | **0.03(0.01)** | **0.030** | **0.07** |
|  | P_T_<0.7 | -0.006(0.01) | 0.66 | na | **0.03(0.01)** | **0.034** | **0.07** |
|  | P_T_<0.9 | -0.006(0.01) | 0.65 | na | **0.03(0.01)** | **0.033** | **0.07** |
|  | P_T_<1 | -0.006(0.01) | 0.65 | na | **0.03(0.01)** | **0.033** | **0.07** |
| 17 y | P_T_<0.001 | 0.001(0.02) | 0.96 | na | **0.039(0.02)** | **0.012** | **0.13** |
|  | P_T_<0.01 | 0.008(0.02) | 0.62 | na | **0.058(0.02)** | **0.00016** | **0.32** |
|  | P_T_<0.05 | 0.006(0.02) | 0.69 | na | **0.06(0.02)** | **0.00010** | **0.34** |
|  | P_T_<0.1 | 0.007(0.02) | 0.64 | na | **0.059(0.02)** | **0.00012** | **0.33** |
|  | P_T_<0.3 | -0.001(0.02) | 0.93 | na | **0.064(0.02)** | **0.000043** | **0.38** |
|  | P_T_<0.5 | -0.001(0.02) | 0.94 | na | **0.066(0.02)** | **0.000022** | **0.41** |
|  | P_T_<0.7 | -0.003(0.02) | 0.85 | na | **0.065(0.02)** | **0.000030** | **0.39** |
|  | P_T_<0.9 | -0.002(0.02) | 0.90 | na | **0.064(0.02)** | **0.000034** | **0.39** |
|  | P_T_<1 | -0.002(0.02) | 0.90 | na | **0.064(0.02)** | **0.000033** | **0.39** |

Polygenic scores for risk-increasing alleles (PGS) were constructed in ALSPAC and then Z-standardised. Rank-transformed SCDC scores (at 8, 11, 14 and 17 years) were regressed on ASD-PGS and schizophrenia-PGS using OLS, and are shown by PGS bin; Adjusted-R^2^ (Adj-R^2^) is reported for *P*<0.05 (indicated in bold); All ALSPAC children with a clinical ASD diagnosis were excluded from the analysis (i.e. up to 36 ALSPAC children)

Unadjusted/Adjusted - OLS regression with ASD-PGS and schizophrenia-PGS adjusted for each other, and unadjusted otherwise; ALSPAC - Avon Longitudinal Study of Parents and Children; ASD - Autism Spectrum Disorders; na - Not available; PGC - Psychiatric Genomics Consortium ; PGC-ASD - ASD collection of the PGC; P_T_ - PGS threshold; OLS - Ordinary Least Square regression; PGC-SCZ2 - Second PGC mega-analysis of SCZ; SCDC - Social-Communication Disorder Checklist; SCZ - Schizophrenia; y - years

**Supplementary Table 9:** Association of ASD and schizophrenia risk scores with SCDC scores (longitudinal analysis of effect changes)

| **PGS bin** | **ASD-PGS(PGC-ASD)**  **PGS x age** | | **Schizophrenia-PGS(PGC-SCZ2)**  **PGS x age** | |
| --- | --- | --- | --- | --- |
|  | **Beta (SE)** | ***P*** | **Beta (SE)** | ***P*** |
| P_T_<0.001 | -0.0011(0.0013) | 0.43 | 0.0009(0.0013) | 0.50 |
| P_T_<0.01 | -0.003(0.0013) | 0.023 | 0.0022(0.0014) | 0.11 |
| P_T_<0.05 | -0.0032(0.0014) | 0.019 | 0.0029(0.0014) | 0.030 |
| P_T_<0.1 | -0.0041(0.0014) | 0.0029 | 0.0029(0.0014) | 0.033 |
| P_T_<0.3 | -0.0051(0.0014) | 0.00018 | 0.0035(0.0014) | 0.011 |
| P_T_<0.5 | -0.0051(0.0013) | 0.00016 | 0.0035(0.0014) | 0.010 |
| P_T_<0.7 | -0.0051(0.0013) | 0.00015 | 0.0033(0.0014) | 0.015 |
| P_T_<0.9 | -0.005(0.0013) | 0.00020 | 0.0032(0.0014) | 0.019 |
| P_T_<1 | -0.005(0.0013) | 0.00021 | 0.0032(0.0014) | 0.018 |

Polygenic scores for risk-increasing alleles (PGS) were constructed in ALSPAC and then Z-standardised (for simplicity only a PGS threshold P_T_<0.05 is shown). Longitudinal measures of untransformed SCDC-score counts were regressed on ASD-PGS and schizophrenia-PGS simultaneously allowing for changes in genetic effects over time (PGS x age interaction).

ALSPAC - Avon Longitudinal Study of Parents and Children; ASD - Autism Spectrum Disorders; PGC - Psychiatric Genomics Consortium; PGC-ASD - ASD collection of the PGC; PGC-SCZ2 - Samples of the second PGC mega-analysis of SCZ; P_T_ - PGS threshold; SCDC - Social-Communication Disorder Checklist; SCZ - Schizophrenia

**Supplementary Table 10:** Association of ASD and schizophrenia risk scores with SCDC-missingness

|  | ***ASD-PGS (PGC-ASD)*** | | | | | | | | |
| --- | --- | --- | --- | --- | --- | --- | --- | --- | --- |
| **SCDC** | **Unadjusted (N_Total_=7,758)** | | | | | **Adjusted (N_Total_=7,301)** | | | |
|  | **N_miss_** | **OR (SE)** | ***P*** | **Ps-R^2^ (%)** | **N_miss_** | | **OR (SE)** | ***P*** | **Ps-R^2^ (%)** |
| 8 y | 2,182 | 0.96(0.02) | 0.090 | na | 1,848 | | 0.99(0.03) | 0.82 | na |
| 11 y | 2,531 | 0.96(0.02) | 0.081 | na | 2,181 | | 0.99(0.03) | 0.67 | na |
| 14 y | 2,907 | 0.95(0.02) | 0.031 | 0.05 | 2,536 | | 0.97(0.02) | 0.22 | na |
| 17 y | 3,744 | 0.96(0.02) | 0.064 | na | 3,352 | | 0.98(0.02) | 0.44 | na |
|  | ***Schizophrenia-PGS (PGC-SCZ2)*** | | | | | | | | |
| **SCDC** | **Unadjusted (N_Total_=7,758)** | | | | | **Adjusted (N_Total_=7,301)** | | | |
|  | **N_miss_** | **OR (SE)** | ***P*** | **Ps-R^2^ (%)** | **N_miss_** | | **OR (SE)** | ***P*** | **Ps-R^2^ (%)** |
| 8 y | 2,182 | 1.11(0.03) | 0.000058 | 0.18 | 1,848 | | 1.12(0.03) | 0.000052 | 0.21 |
| 11 y | 2,531 | 1.11(0.03) | 0.000037 | 0.17 | 2,181 | | 1.11(0.03) | 0.000036 | 0.20 |
| 14 y | 2,907 | 1.09(0.03) | 0.00020 | 0.14 | 2,536 | | 1.1(0.03) | 0.00018 | 0.15 |
| 17 y | 3,744 | 1.1(0.03) | 0.000050 | 0.15 | 3,352 | | 1.1(0.03) | 0.000050 | 0.17 |

Polygenic scores (PGS) for risk-increasing alleles (PGS) were constructed in ALSPAC and then Z-standardised (for simplicity only a PGS threshold P_T_<0.05 is shown). SCDC-missingness was regressed on ASD-PGS and schizophrenia-PGS using logistic regression. Odds ratios (OR) and their standard errors are given per one-standard-deviation increase in PGS (at 8, 11, 14 and 17 years) in ALSPAC one-year survivors; Ps-R^2^ (McFadden pseudo-R^2^) is reported for *P*<0.05; Unadjusted/Adjusted - With and without adjustment for maternal education; ALSPAC - Avon Longitudinal Study of Parents and Children; ASD - Autism Spectrum Disorders; na - Not available; PGC - Psychiatric Genomics Consortium ; PGC-ASD - ASD collection of the PGC; P_T_ - PGS threshold; PGC-SCZ2 - Second PGC mega-analysis of SCZ; SCDC - Social-Communication Disorder Checklist; SCZ - Schizophrenia; y - years; N_Total_ - Total N; N_miss_ - Number of dropouts

**Supplementary figures**

**
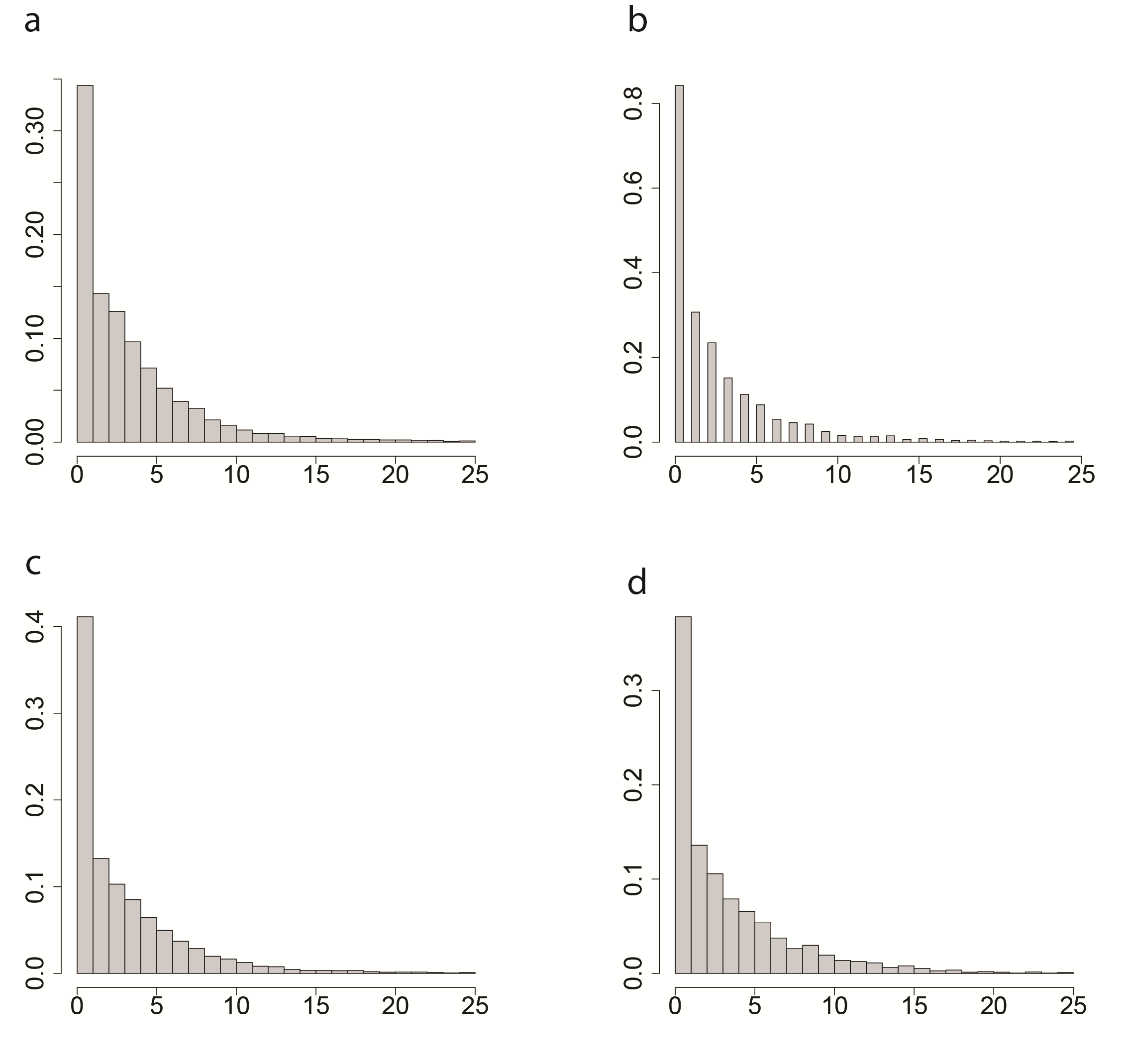
**

**Supplementary Figure 1:** Distribution of raw SCDC scores in ALSPAC

a - 8 years, b - 11 years; c - 14 years and d - 17 years

ALSPAC - Avon Longitudinal study of Parents and Children; SCDC - Social-Communication Disorder Checklist


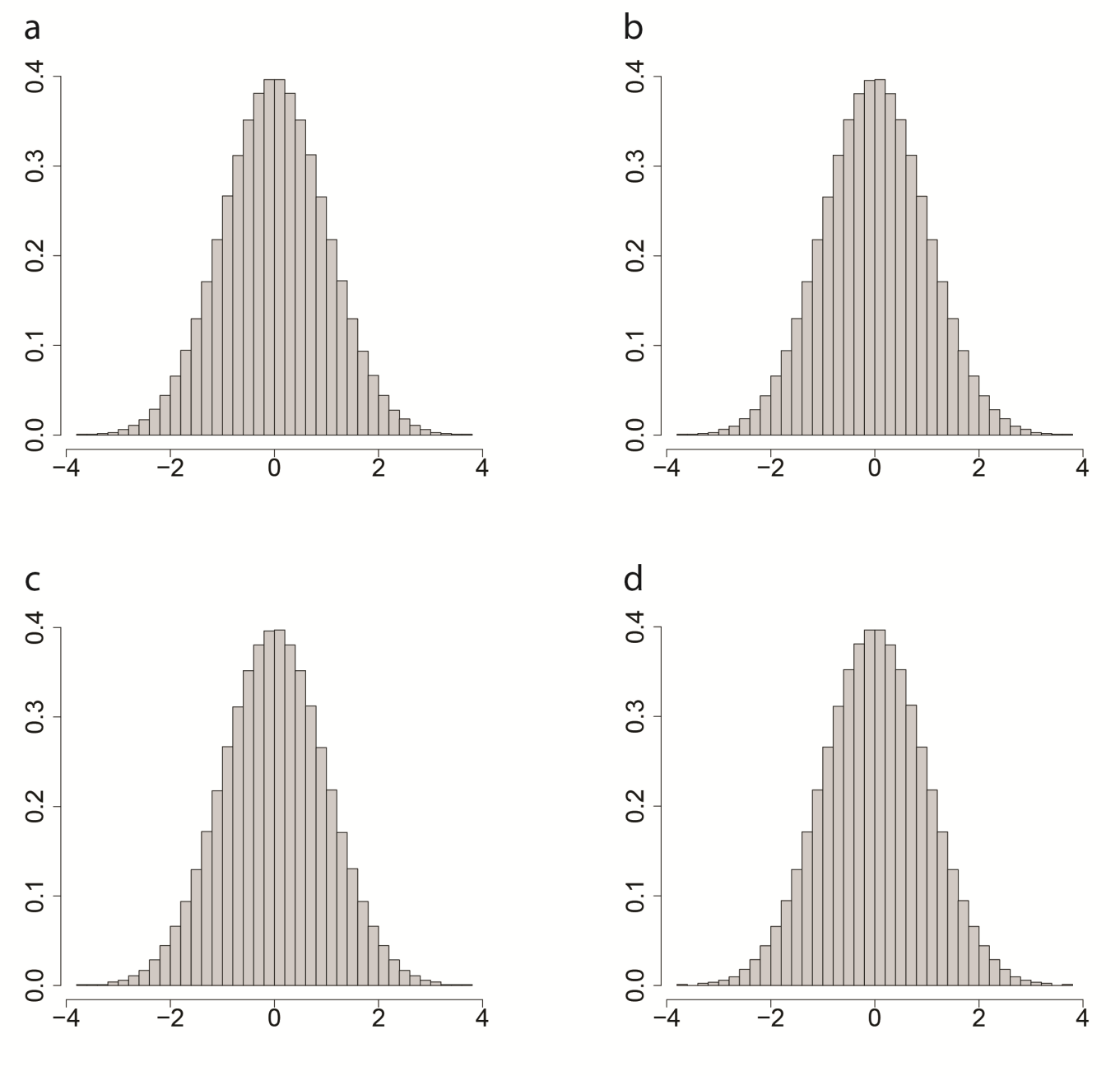


**Supplementary Figure 2:** Distribution of rank-transformed SCDC scores in ALSPAC

a - 8 years, b - 11 years; c - 14 years and d - 17 years

ALSPAC - Avon Longitudinal study of Parents and Children; SCDC - Social-Communication Disorder Checklist
